# Genetic contributions to the etiology of anorexia nervosa: New perspectives in molecular diagnosis and treatment

**DOI:** 10.1002/mgg3.1244

**Published:** 2020-05-05

**Authors:** Stefano Paolacci, Aysha Karim Kiani, Elena Manara, Tommaso Beccari, Maria Rachele Ceccarini, Liborio Stuppia, Pietro Chiurazzi, Laura Dalla Ragione, Matteo Bertelli

**Affiliations:** ^1^ MAGI'S LAB Rovereto Trento Italy; ^2^ MAGI EUREGIO Bolzano Italy; ^3^ Department of Pharmaceutical Sciences University of Perugia Perugia Italy; ^4^ Department of Psychological, Health and Territorial Sciences School of Medicine and Health Sciences "G. d'Annunzio" University Chieti Italy; ^5^ Istituto di Medicina Genomica Università Cattolica del Sacro Cuore Rome Italy; ^6^ UOC Genetica Medica Fondazione Policlinico Universitario “A. Gemelli” IRCCS Rome Italy; ^7^ Center for the Treatment of Eating Disorders Residenza Palazzo Francisci Todi Perugia Italy; ^8^ EBTNA‐LAB Rovereto Trento Italy

**Keywords:** Anorexia nervosa, Genetic test, Genetic variant

## Abstract

**Background:**

Anorexia nervosa is a multifactorial eating disorder that manifests with self‐starvation, extreme anxiety, hyperactivity, and amenorrhea. Long‐term effects include organ failure, disability, and in extreme cases, even death.

**Methods:**

Through a literature search, here we summarize what is known about the molecular etiology of anorexia nervosa and propose genetic testing for this condition.

**Results:**

Anorexia nervosa often has a familial background and shows strong heritability. Various genetic studies along with genome‐wide association studies have identified several genetic loci involved in molecular pathways that might lead to anorexia.

**Conclusion:**

Anorexia nervosa is an eating disorder with a strong genetic component that contributes to its etiology. Various genetic approaches might help in the molecular diagnosis of this disease and in devising novel therapeutic options.

## Abstract 2

Anorexia nervosa is a multifactorial eating disorder with a strong genetic component that manifests with self‐starvation, extreme anxiety, hyperactivity, and amenorrhea. Through a literature search, here we summarize what is known about the molecular etiology of anorexia nervosa and propose genetic testing for this condition.

## 1 INTRODUCTION

Stable body weight maintenance is one of the key elements of human survival, which is achieved by the balance of intake and expenditure of energy. To keep this balance, control of food intake is a vital factor that involves a complex system of central as well as peripheral physiological signals (Abdalla, 2017).

According to the fifth edition of the Diagnostic and Statistical Manual of Mental Disorders (DSM‐V), anorexia nervosa (AN) is defined as a disorder in which patients weigh less than minimally normal (adults) and less than minimally expected (children and adolescents) in the context of age, sex development, and physical health, and show persistent behavior that interferes with weight gain (Clarke, Weiss, & Berrettini, 2012).

In acute cases, usual symptoms of AN patients include fatigue, dizziness, and syncope, while in chronic patients, all organs are affected because of malnutrition (Zipfel, Giel, Bulik, Hay, & Schmidt, 2015).

AN is a complex and serious illness, affecting 0.9%–4% of women and 0.3% of men, with twin‐based heritability estimates of 50%–60%. The onset of the disease occurs mostly during adolescence, though onset before puberty is also not unusual, and women are sometimes diagnosed with AN at midlife or older adulthood (Watson et al., 2019).

AN has the highest mortality rate among psychiatric disorders because it can result in significant medical complications (Moskowitz & Weiselberg, 2017). A meta‐analysis reported a rough mortality rate of 5.1 deaths per 1,000 individuals per year (Arcelus, Mitchell, Wales, & Nielsen, 2011).

AN is a multifactorial disorder, but there is a strong genetic component. For instance, it is known that those consanguineous of AN patients are more likely to have AN than the relatives of unaffected controls (Strober, Freeman, Lampert, Diamond, & Kaye, 2000). Furthermore, twin and linkage studies indicate that genetic factors play a fundamental role (Devlin, Jones, Bacanu, & Roeder, 2002; Klump, Miller, Keel, McGue, & Iacono, 2001; Kortegaard, Hoerder, Joergensen, Gillberg, & Kyvik, 2001).

Treatment of AN is difficult, which accounts for the high morbidity and mortality of AN. Medication options are still under trial and research is going on to identify drugs that can target the pathway associated with this disorder.

The aim of this review is to summarize the present knowledge on the molecular and genetic determinants of AN through literature search, and to propose genetic testing for AN that includes those genes that might carry rare variants predisposing to AN and genes that, when mutated, cause syndromic conditions in which anorexia is one of the features.

## 2 MOLECULAR PATHWAYS INVOLVED IN ANOREXIA NERVOSA

Among all species, food intake is a conserved behavioral characteristic that involves various biological systems, such as the serotonergic pathway, which is a conserved neural system that controls the feeding behavior of humans and other mammals, such as rodents.

### 2.1 Serotonin pathway

Serotonin or 5‐hydroxytryptamine (5‐HT) receptors are crucial for the regulation of molecular substrates required for survival, such as food intake, prevention of depressive states, control of anxiety, as well as fear of novelty, learning, memory, locomotion, and peripheral functions (i.e., gastrointestinal peristalsis). Any dysregulation in the 5‐HT systems results in symptoms similar to AN. The reuptake of 5‐HT was observed to be increased by the administration of estrogen, which alters the mRNA as well as the protein levels of various serotonin markers and reduces the breakdown of 5‐HT. Persistent external stress could limit neuronal plasticity associated with the serotonin pathway and predispose to anorexia (Compan, 2017; Lokuge, Frey, Foster, Soares, & Steiner, 2011). Similarly, AN patients might also show abnormalities in the *HTR1D* (serotonin 1D) gene (OMIM *182133) (Brown et al., 2007).

### 2.2 Opioid and dopamine pathways

Opioid peptides are derived from pro‐opiomelanocortin maturation and help regulate the balance between energy intake and expenditure through reward‐mediated behavior. Opioid peptides are expressed in the paraventricular nucleus of the hypothalamus, ventromedial hypothalamus, amygdala, nucleus accumbens, and the forebrain regions (Hasan & Hunaid, 2011).

Dopamine is a neuromodulating catecholamine found throughout the mammalian central nervous system, and midbrain dopamine‐containing neurons regulate emotional behavior, natural motivation, reward, and cognitive function (Chao & Nestler, 2004).

Several studies show that AN might be caused by an altered dopamine pathway. For instance, patients with AN have low levels of the major dopamine metabolite, homovanillic acid, in their cerebrospinal fluid (Kaye, Ebert, Gwirtsman, & Weiss, 1984).

According to another study, dieting in association with high levels of exercise promotes an increase in dopamine, that in turn facilitates rewarding behaviors such as diet and exercise, that can become habits similar to drug dependency or self‐starvation (Södersten, Nergårdh, Bergh, Zandian, & Scheurink, 2008). Abnormalities in the dopamine pathway can also cause hyperactive motor behavior along with behavioral inhibition (Klenotich, Ho, McMurray, Server, & Dulawa, 2015; Moskowitz & Weiselberg, 2017).

### 2.3 Vitamin D3 as a neurosteroid

Vitamin D3 is a steroid hormone recognized to have many extra‐skeletal roles, and serum vitamin D3 deficiency has been correlated to obesity and diabetes. It is known that vitamin D3 modulates peroxisome proliferator‐activated receptor gamma (PPARγ), involved in inflammation related to the diet (Tasegian et al., 2016). A cross‐sectional study was done to meta‐analyze the vitamin D3 parameters in AN patients relative to healthy controls. Results showed that the metabolite of vitamin D3 (25‐hydroxyvitamin D), as well as vitamin D3 levels of AN patients, were significantly lower without proper supplementation (Giollo et al., 2017; Veronese et al., 2015).

Patients with AN have a vitamin D3 deficiency that leads to defects in bone mineralization. Furthermore, vitamin D3 can also play a role as a neurosteroid. The connection between vitamin D3 and AN might be its ability to regulate neurotrophic factors, provide neuroprotection, modulate neurotransmission, and contribute to synaptic plasticity. Studies in rats showed that vitamin D3 is present already in the early phases of brain development. It is interesting to note that the initial expression of the vitamin D receptors in the rat brain corresponds with the appearance of dopaminergic neurons within the mesencephalon, but the vitamin D receptors are also expressed within the dopaminergic neurons in the adult substantia nigra (Groves, McGrath, & Burne, 2014).

It has been observed that mice lacking vitamin D receptor have increased anxiety (Kalueff, Lou, Laaksi, & Tuohimaa, 2004); this is consistent with the role of vitamin D3 in the repression of serotonin reuptake, transport, and degradation. The diminished brain levels of serotonin and vitamin D3 could result in social behavior changes associated with autism spectrum disorders and depression (Sabir et al., 2018). Finally, vitamin D3 role in AN might be associated with the increase in the expression of genes involved in estrogen biosynthesis. In fact, vitamin D3 is a powerful regulator of sex steroid hormone production in porcine granulosa cells via the modulation of steroidogenic enzymes (Hong et al., 2017).

### 2.4 Appetite‐regulating hormones

The regulation of body weight relies on both central and peripheral stimuli. The central regulation of appetite is due to the hypothalamus, which integrates signals coming from the periphery. Although some variants that affect genes expressed in the hypothalamus have been linked to obesity, it is also known that lesions in the lateral hypothalamus can cause a dramatic reduction of food intake in rats (Anand & Brobeck, 1951).

The central part of the hypothalamus (the arcuate nucleus) integrates signals regarding food intake and energy expenditure (Miller, 2017). This regulation is based on the expression of neuropeptides and their receptors. Other signals that participate in the regulation of energy intake and expenditure come from the periphery of the organism and can be produced by the adipose tissue, such as leptin, or from the gut, such as cholecystokinin (CCK) and ghrelin (Miller, 2017).

The gut has specific endocrine cells that release various hormones in response to nutrient intake. Postprandial satiety is regulated by the communication between gut and hypothalamus, involving appetite hormones. These appetite modulators comprise both orexigenic and anorexigenic hormones, such as ghrelin, leptin, CCK, peptide YY (PYY), pancreatic polypeptide (PP), oxyntomodulin (OXM), and glucagon‐like peptide (GLP)‐1. After stimulation, anorexigenic peptides are released while the levels of the orexigenic peptide ghrelin reduce (Perry & Wang, 2012).

Ghrelin is produced in the stomach and pancreatic cells and exhibits a wide range of effects on feeding behavior, reward mechanisms, reproduction, and growth, and results in positive energy balance (Perry & Wang, 2012). Ghrelin has a link with AN. This is evident from numerous animal studies in which ghrelin activates the reward pathways in the brain, which in turn contribute to AN and other addictive disorders, such as alcoholism (Perello & Dickson, 2015).

Leptin is produced by adipocytes and exhibits a powerful effect on food intake and energy expenditure. Leptin acts both peripherally and centrally by reducing the appetite and creating an overall negative energy balance (Steiner & Romanovsky, 2007). In a study by Monteleone et al. (2005), the serum level of leptin was reported to be significantly decreased in AN patients but only moderately increased in obese patients. In the hypothalamus, leptin acts through cell surface receptors, mediated by feeding regulators like neuropeptide Y (NPY) (Palmiero Monteleone et al., 2005).

NPY is a member of the pancreatic polypeptide family, and its levels reflect the nutritional status of the body, and during fasting, NPY levels increase, then decrease after re‐feeding (Beck, 2006).

The pancreatic polypeptide (PPY) is secreted postprandially, and its peripheral administration decreases appetite along with weight loss through inhibition of the arcuate hypothalamic nucleus expression of NPY/AGRP (De Silva & Bloom, 2012).

Similarly, CCK acts with the help of its receptor and causes short‐term food intake inhibition (Cummings & Overduin, 2007), and GLP‐1 increases satiety and decreases food intake (Shah & Vella, 2014). OXM, which acts through GLP‐1 receptor, causes short‐term food intake suppression and reduction of plasma levels of ghrelin (Perry & Wang, 2012).

### 2.5 Endocannabinoid pathway

The endocannabinoid system plays a significant role in the regulation of appetite. This endogenous cannabinoid neuromodulator system includes two cannabinoid receptors, CB1 and CB2, along with endogenous ligands like anandamide (N‐arachidonoylethanolamine), 2‐arachidonoylglycerol, as well as the enzymes contributing to their synthesis or degradation, such as monoacylglycerol lipase (MAGL), fatty acid amide hydrolase (FAAH), N‐acyl phosphatidylethanolamine phospholipase D (NAPE‐PLD), and diacylglycerol lipase (DAGL) (Berry & Mechoulam, 2002; Doris, Millar, Idris, & O’Sullivan, 2019). Animal models showed that endocannabinoids enhance feeding; in fact, the blockade of cannabinoid receptor CB1 suppresses eating (Hao, Avraham, Mechoulam, & Berry, 2000). The CB1 knockout (KO) mice eat less than their normal littermates after starvation (Di Marzo et al., 2001). Furthermore, cannabinoids are known to induce “reward” effects, such as the feeling of pleasure after eating tasty food (Di Marzo et al., 2001).

Genetic variants in the *CNR1* (OMIM *114610) gene, which encodes the CB1 (cannabinoid 1) receptor, might contribute significantly to the susceptibility to AN. This hypothesis of the potential association of *CNR1* variants with AN was further investigated after the identification in this gene of a triplet repeat marker in a family‐based study (Siegfried et al., 2004). The involvement of *CNR1* trinucleotide repeats might be one of the explanations for the non‐Mendelian inheritance of AN, though more functional studies are needed to prove the differential effect of the various (AAT)_n_ repeats on the CB1 receptor.

The role of the cannabinoid receptors is also supported by studies performed on anandamide (also known as N‐arachidonoylethanolamine), a fatty acid neurotransmitter derived from the arachidonic acid and degraded by the FAAH enzyme. In particular, one of the effects of anandamide might be the modulation of neuronal structure. In fact, by binding to CB1R, anandamide inhibits neuronal differentiation and causes the retraction of neurites (Rueda, Navarro, Martínez‐Serrano, Guzmán, & Galve‐Roperh, 2002). The differentiation, induced by nerve growth factor (NGF), of PC12 cells (expressing the anandamide receptor CB1) was inhibited by anandamide administration, which blocks the activation of the NGF receptor. Thus, anandamide plays a key role in the establishment of synaptic plasticity (Goodfellow & Glass, 2009). This reduction of neural plasticity caused by anandamide might explain why levels of anandamide are significantly enhanced in the plasma of anorexic women. Moreover, circulating anandamide levels were significantly and inversely correlated with plasma leptin concentrations in both healthy controls and anorexic women (Palmiero Monteleone et al., 2005).

Palmitoylethanolamide (PEA) is an endogenous fatty acid amide. The main target of PEA is the peroxisome proliferator‐activated receptor α (PPARα). PEA can also bind to cannabinoid‐like G‐coupled receptors GPR55 and GPR119. The anorectic action of exogenous PEA is mediated by the activation of the transcription factor PPARα in the small intestine (Hansen, Kleberg, & Hassing, 2015). In mouse, intestinal concentration of PEA decreases in response to high‐fat feeding, and it was speculated that PEA might function as a biosensor for dietary fat. PEA, after the activation of PPARα, brings the signal further through the vagus nerve to the brain (Di Guida, 2017). In underweight AN patients, plasma PEA concentration increases after exposure to a non‐favorite meal and progressively decrease after eating it, whereas plasma concentrations of PEA progressively decrease in hedonic eating (Monteleone et al., 2015).

## 3 GENETICS OF ANOREXIA NERVOSA

AN has a strong genetic component. It sometimes has a familial presentation; there is a fourfold increase in susceptibility to AN among family members, and female relatives of AN patients have an 11 times greater risk of disease compared to the normal population (Zipfel et al., 2015). Studies have shown that genes contribute more than 50% to 74% of AN developing risk (Moskowitz & Weiselberg, 2017), and monozygotic twins have a higher likelihood of developing AN then dizygotic twins (Yilmaz, Hardaway, & Bulik, 2015). Generally, when AN cases are defined using strict criteria, the heritability is higher compared to the heritability of broadly defined “subsyndromic” AN cases.

### 3.1 Rare variants with Mendelian segregation

In one recent study, using whole‐exome analysis in two independent families with male individuals with AN, the authors found variants in the neuronatin (*NNAT*) gene (OMIM *603106) in both probands: one nonsense variant (p.Trp33*) and one rare variant in the 5′UTR, respectively (Lombardi et al., 2019). Afterward, to confirm their data, a screening of the *NNAT* was conducted in a cohort of eight male and 144 female individuals with AN, and a further 11 *NNAT* variants were found, showing that 40% and 6% of male and female AN individuals carried an *NNAT* variant, respectively. The protein encoded by *NNAT* is a proteolipid that might be involved in the regulation of ion channels during brain development. The encoded protein might also participate in the maintenance of segment identity in the hindbrain and pituitary development (Lombardi et al., 2019).

In a study published in 2014 (Scott‐Van Zeeland et al., 2014), the authors analyzed a series of 152 candidate genes involved in feeding behaviors, dopamine function, serotonin signaling, and genes previously associated using GWAS, such as *OPRD1* (OMIM *165195) and *EPHX2* (OMIM *132811), to identify genetic variants that contribute to AN. DNA sequencing was performed in a cohort of about 700 AN, and rare *EPHX2* variants were identified as significantly associated with AN (Scott‐Van Zeeland et al., 2014).

The *EPHX2* encodes an epoxide hydrolase found in both the cytosol and peroxisomes and involved in cholesterol metabolism. The protein is expressed in neural tissues relevant to AN and *EPHX2* transcription might be induced by sex hormones and is regulated by the hypothalamic‐pituitary‐gonadal axis. This would account for the potential role of this gene in the etiology or maintenance of AN. Many studies have reported high serum cholesterol levels in malnourished individuals suffering from AN (Scott‐Van Zeeland et al., 2014). In the same study, the authors reported that *ESR2* (OMIM *601663), encoding the estrogen receptor 2, might harbor variants associated with AN. This association is interesting because most of the AN cases are female. Additionally, these results are consistent with previous research suggesting that estrogens and their receptor are linked with the etiology of AN (Scott‐Van Zeeland et al., 2014).

Another study combined exome sequencing, whole‐genome sequencing, and linkage analysis to examine two families with recurrence of eating disorders, especially AN. In the first pedigree, the best candidate to explain the predisposition to eating disorders was a missense variant co‐segregating with the affected family members in the *ESRRA* (estrogen‐related receptor alpha) (OMIM *601998), while a potentially damaging mutation in the *HDAC4* (histone deacetylase 4) (OMIM *605314) was reported in the second pedigree (Cui et al., 2013). These genes play a significant role in the estrogen system. Thus, this would explain why there is a majority of affected female family members. *ESRRA* encodes a nuclear receptor that has sequence homology with estrogen receptors and plays a role in energy balance as well as metabolism. *HDAC4* encodes a histone deacetylase and is also linked to the development and function of central nervous system. Further transcriptional studies revealed that expression of the HDAC4 deacetylase repressed the transcription of ESRRA‐induced target genes, whereas ESRRA and HDAC4 exhibited interaction in both in vivo and in vitro studies. These results suggest that the identified variants cause a decrease in the activity of ESRRA and an increase in the likelihood of developing AN (Sild & Booij, 2019).

In a 2017 study, a whole‐exome sequencing approach was used to analyze 38 individuals with restricting AN and 55 individuals with AN associated with binge/purge behaviors (Lutter et al., 2017). The genes carrying predicted damaging variants mainly belonged to three pathways: (a) neuropeptide hormone signaling, (b) inflammatory pathway, and (c) cholinergic neurotransmission. Consistently, most of the identified neuropeptides regulate appetite and body weight homeostasis. An in silico approach then evaluated the pathogenicity of the identified variants. One variant in *NTS* (OMIM *162650), the gene encoding the neuropeptide transmitter neurotensin, disrupts a motif required for the generation of mature neuropeptide neurotensin through cleavage. Three other *NTS* variants occur in the portion of the propeptide that is maturated to generate neuromedin N, which also binds the neurotensin receptor. Furthermore, the authors found one individual who harbored a truncating variant in the neurotensin receptor 1 (*NTSR1*) gene (OMIM *162651). Of note, this variant co‐segregated with the AN phenotype in the daughter and granddaughter (Lutter et al., 2017).

Finally, 245 genes were identified with damaging variants at a significantly higher rate than predicted in the binge‐eating AN cohort. *UNC80* (OMIM *612636) had the most damaging variants in the binge‐eating group; the encoded protein is a component of the nonselective sodium leak channel (NALCN), an ion channel regulated by the peptide neurotransmitter substance P and neurotensin. Another important neuropeptide, preproglucagon (synthesized from the *GCG*, OMIM *138030), has been found to carry two variants. This protein is proteolytically cleaved to generate several neuropeptides in the brain. The first variant in *GCG* eliminates an arginine required for the generation of oxyntomodulin, while the second variant replaces an arginine within the cleavage site required for generation of GLP‐1. Both oxyntomodulin and GLP‐1 are agonists for the GLP‐1 receptor. Finally, the authors also detected predicted damaging variants in both *BDNF* (OMIM *113505) and its receptor, *NTRK2* (OMIM *600456). Of note, *BDNF* and *NTRK2* have previously been implicated in food intake and body weight regulation (Lutter et al., 2017).

Two very recent articles by Bienvenu et al. in 2019 support the multigenic etiology of AN. In the first study, the authors sequenced the whole exome of one family and found three ultra‐rare deleterious variants within the *DRD4* (OMIM *126452), *NMS*, and *CCKAR* (OMIM *118444) (Bienvenu et al., 2019b). *DRD4* encoded a dopamine receptor and carried the p.Leu356Arg rare variant in the highly conserved region of one of its intracellular loops in all three affected members of the family. *NMS* encodes a 36‐amino acid pre‐proprotein (neuromedin S) that is proteolytically processed to generate a neuropeptide that plays a role in the regulation of anorexigenic action, and stimulation of oxytocin and vasopressin release. This protein carried a p.Met1Thr variant in all affected members. Finally, *CCKAR*, which encodes a CCK‐binding receptor, had a rare variant, p.Cys387Tyr, in the cytoplasmic C‐terminal region that segregated in all the affected individuals. Thus, the co‐segregation of these three variants in genes linked with the reward pathway, in three affected members of the same family, suggests a predisposing role of this variant in the onset of AN (Bienvenu et al., 2019b).

In the other study, the authors, following whole‐exome sequencing in nine female AN individuals and their parents, found seven de novo variants. Of them, four are present in genes and participate in the dopamine pathway and neuron differentiation: *CSMD1* (OMIM *608397), *CREB3* (OMIM *606443), *PTPRD* (OMIM *601598), and *GAB1* (OMIM *604,439) (Bienvenu et al., 2019a).

### 3.2 Genome‐wide and candidate genes association studies

Both genome‐wide (GWAS) and candidate genes association studies have revealed the genetic loci contributing to the etiology of AN.

The first two genome‐wide association studies identified an AN‐susceptibility locus on chromosome 1 (Grice et al., 2002; Nakabayashi et al., 2009). Further studies have identified these AN loci as opioid delta 1 receptor (OPRD1), which acts as a receptor for endogenous opioids, and serotonin 1D (HTR1D), which is a 5‐HT receptor and acts on central nervous system and affects anxiety as well as locomotion (Bergen et al., 2003; Brown et al., 2007).

For the further advancement and exploration of genomic study as well as genetic correlations in AN, Watson et al. conducted a genome‐wide association study including data from the Anorexia Nervosa Genetic Initiative (ANGI), the Genetic Consortium for Anorexia Nervosa (GCAN), and the Wellcome Trust Case Control Consortium‐3 (WTCCC‐3) along with UK Biobank, thus comprising 16,992 cases as well as 55,525 controls from 17 European countries. This identified eight loci encompassing the genes *NCKIPSD* (OMIM *606671), *CADM1* (OMIM *605686), *ERLEC1* (OMIM *611229), *MGMT* (OMIM *156569), *FOXP1* (OMIM *605515), *CDH10* (OMIM *604555), *PTBP2* (OMIM *608449), and *NSUN3* (OMIM *617491), which carry polymorphisms that significantly correlate with AN and metabolic/anthropometric phenotypes (Watson et al., 2019).

Furthermore, according to the Gene Ontology (GO) project, one of the molecular pathways that positively regulates embryonic development and that might be associated with AN principally involves two genes, *DAG1* (OMIM *128239) and *CTNNB1* (OMIM *116806). *DAG1* encodes dystroglycan 1, a protein that plays an important role in connecting the extracellular matrix with the cytoskeleton, while *CTNNB1* encodes catenin β‐1, a protein that constitutes the adherens junctions and has a role in Wnt signaling (Bello et al., 2015).

Another GWAS including 1,033 AN cases and 3,733 controls identified new genetic risk factors. The results of this GWAS, besides confirming the association of *OPRD1* and *HTR1D*, identified novel risk loci that showed a marginally significant association with AN: *NTNG1* (OMIM *608818), *LRP2* (OMIM *600073), *CDH9* (OMIM *609974), *ZNF804B*, *VGLL4* (OMIM *618692), and *AKAP6* (OMIM *604691) (Wang et al., 2011).

Finally, another GWAS confirmed the association of the *EBF1* (OMIM *164343), a regulator of the adipocyte lineages development, that, when knocked out in mice, causes a reduction of adipose tissue and decrease of circulating leptin (Li et al., 2017).

The involvement of serotonergic genes in the etiology of AN was investigated by candidate gene studies. For instance, the polymorphism 5‐HTTLPR (serotonin transporter‐linked polymorphic region) is a degenerate repeat polymorphic region upstream of the coding region of the *SLC6A4* (OMIM *182138), with alleles classified as long and short based on the number of repeats. A study found that the short allele of 5‐HTTLPR is significantly associated with the increased risk of AN onset in a Chinese cohort (Chen et al., 2015). In another case‐control study performed in a Mexican population, the authors found that the rs6311 polymorphism in the *HTR2A* (OMIM *182135) gene is significantly associated with eating disorders, and individuals who are homozygous for the alternate allele have an increased risk for suicide attempts as comorbidity (Genis‐Mendoza et al., 2019). This result was separately confirmed in an Italian cohort (Ceccarini et al., 2019).

As mentioned above, the dopaminergic pathway regulates feeding behavior, motor activity, and reward‐motivated behavior (Chao & Nestler, 2004). Thus, it is plausible that variants in the dopamine receptors might play a role in the onset of AN. In 2005, an analysis conducted in an AN cohort of polymorphisms in the *DRD2* (OMIM *126450) gene, encoding the dopamine 2 receptor, revealed that single‐nucleotide polymorphisms (SNPs) were not significantly associated with AN. However, when those SNPs were considered as a haplotype, their association with AN became significant (Bergen et al., 2005).

*BDNF* (encoding the brain‐derived neurotrophic factor) is another extensively studied candidate gene for the risk of AN. An extensive family‐based study of eight European populations consisting of 453 eating disorders (ED) trios reported an association of the Val66Met (rs6265) polymorphism with restricting subtype of AN and low minimum body mass index (BMI) (Ribasés et al., 2005). According to other genetic studies, there is a 30% higher incidence of eating disorders among individuals with the Val/Met and the Met/Met polymorphism of BDNF (Gratacòs et al., 2007; Mercader et al., 2007). Moreover, in a recent study carried out in an Italian cohort composed of 556 ED patients and 355 negative controls, the authors demonstrated a strong association between the Met/Met genotype in AN subjects compared with the control samples (Ceccarini et al., 2019).

The polymorphism rs5030980 in the Agouti‐related protein gene (*AGRP*, OMIM *602311) that encodes an antagonist of the melanocortin‐3 and melanocortin‐4 receptors is associated with AN, whereas another SNP, rs13338499, is associated with minimum BMI in 745 AN patients (Dardennes et al., 2007).

The endocannabinoid system, comprising of CB1 and CB2 receptors, contributes to the regulation of appetite and other physiological pathways linked to AN. Genetic variants in *CNR1*, such as the rs1049353 SNP, have been suggested to play a significant role in AN etiology. These findings suggest that endocannabinoids might have a fundamental role in the regulation of stable energy balance and the food intake system (Monteleone et al., 2009; Wirz, Reuter, Felten, & Schwabe, 2018). Several studies have reported an overrepresentation of variants in the cannabinoid receptor 1 (CNR1) in patients with AN, but these case‐control studies need to be replicated (Monteleone et al., 2009). One of these studies comprised 52 family trios with single female AN patients, and four families with two affected individuals. Genetic analysis of these families revealed that the restricting and bingeing/purging subtypes of AN are associated with the eight alleles (AAT7, AAT9–15) of AAT trinucleotide repeat located downstream the coding region of the *CNR1* (Siegfried et al., 2004). Another candidate gene study revealed that the microduplication at the 15q11.2 BP1 BP2 locus gives susceptibility to AN. This locus was analyzed in 1017 AN cases and 7250 controls and the results showed that *NIPA1* is one of the four non‐imprinted genes of the 15q11.2 BP1 BP2 region with the highest level of expression in the brain. *NIPA1* (OMIM *608145) encodes a magnesium transporter protein found in endosomes and neurons. Interestingly, only microduplications of this region are associated with AN, whereas microdeletions of the same region cause excessive eating (Chang et al., 2019).

The *FAAH* (OMIM *602935) encodes the fatty acid amide hydrolase, a catabolic enzyme necessary for endocannabinoids maturation. The *FAAH* c.385C>A SNP rs324420 elevates endocannabinoid signaling by decreasing the steady‐state levels of the FAAH protein that, in turn, leads to increased anandamide levels (Dincheva et al., 2015). Monteleone et al. investigated the role of FAAH in 134 AN patients and 148 controls and confirmed that the “A” allele of rs324420 is strongly associated with a significant risk of developing AN (Monteleone et al., 2009). See Table 1 for a summary of the SNPs found to be associated with AN.

**Table 1:** SNPs associated with AN

Gene	Protein	SNP	Reference
*BDNF*	Brain‐derived neurotrophic factor	rs6265	Ribasés et al. (2005)
*CNR1*	Endocannabinoid receptor 1	rs1049353	(Monteleone et al., 2009; Wirz et al., 2018)
*DRD2*	Dopamine receptor D2	rs6275, rs6277, rs6278, rs1799732	Bergen et al. (2005)
*DRD4*	Dopamine receptor D4	rs1404956473	Bienvenu et al. (2019b)
*AGRP*	Agouti‐related protein	rs5030980, rs13338499	Dardennes et al. (2007)
*ESR2*	Estrogen receptor‐β	rs1256066, rs944050	Scott‐Van Zeeland et al. (2014)
*EPHX2*	Epoxide hydrolase 2	rs2291635, rs1126452, rs59039594, rs1042032, rs1042064, rs4149259	Scott‐Van Zeeland et al. (2014)
*FAAH*	Fatty acid amide hydrolase	rs324420	Monteleone et al. (2009)
*HTR1D*	Serotonin receptor	rs674386, rs856510, rs652783, rs604030, rs7532266	Brown et al. (2007)
*EBF1*	EBF transcription factor 1	rs11953630, rs9313772	Li et al. (2017)
*HTR2A*	Serotonin receptor 2A	rs6311	Genis‐Mendoza et al. (2019)
*SLC6A4*	Serotonin transporter	rs25531	Chen et al. (2015)
*OPRD1*	Opioid receptor delta 1	rs569356, rs204047, rs204055, rs2298896, rs521809, rs4654327, rs533123	Scott‐Van Zeeland et al. (2014)
*ZNF804B*	Zinc finger protein 804B	rs6959888	Wang et al. (2011)
*LRP2*	LDL receptor‐related protein 2	rs830998	Wang et al. (2011)
*CDH9*	Cadherin 9	rs4479806	Wang et al. (2011)
*VGLL4*	Vestigial like family member 4	rs6782029	Wang et al. (2011)
*AKAP6*	A‐kinase anchoring protein 6	rs2383378	Wang et al. (2011)
*NTNG1*	Netrin G1	rs10494067	Wang et al. (2011)
*NCKIPSD*	NCK‐interacting protein with SH3 domain	rs9821797	Watson et al. (2019)
*CADM1*	Cell adhesion molecule 1	rs6589488	Watson et al. (2019)
*ERLEC1*	Endoplasmic reticulum lectin 1	rs2287348	Watson et al. (2019)
*MGMT*	Methylated O6‐methylguanine‐DNA methyltransferase	rs2008387	Watson et al. (2019)
*FOXP1*	Forkhead box protein P1	rs9874207	Watson et al. (2019)
*PTBP2*	Polypyrimidine tract‐binding protein 2	rs10747478	Watson et al. (2019)
*CDH10*	Cadherin 10	rs370838138	Watson et al. (2019)
*NSUN3*	NOP2/Sun RNA methyltransferase 3	rs13100344	Watson et al. (2019)
*ESRRA*	Estrogen‐related receptor alpha	rs200039730	Cui et al. (2013)
*HDAC4*	Histone deacetylase 4	rs61754648	Cui et al. (2013)
*NMS*	Neuromedin S	rs76201870	Bienvenu et al. (2019b)
*GPR55*	G‐protein‐coupled receptor 55	rs3749073	Ishiguro et al. (2011)

### 3.3 Syndromes with anorexia

Anorexia can also be present in a syndromic context. In OMIM, there are five syndromes with anorexia as a feature in the clinical synopsis.

Sarcoidosis (OMIM #181000) is a disease that mainly involves the lungs, skin, or lymph nodes, and less commonly eyes, liver, heart, and brain. Sarcoidosis is characterized by the abnormal accumulation of inflammatory cells (granulomas) around those organs. Variant haplotypes in *HLA‐DRB1* (OMIM *142857) can confer susceptibility to this syndrome. In particular, the HLA‐DRB1*1101 haplotype is associated with sarcoidosis. Phe47 is the amino acid residue most associated with sarcoidosis. This residue is found in the three haplotypes most associated with sarcoidosis (HLA‐DRB1*1101, HLA‐DRB1*1201, and HLA‐DRB1*1501) and might have a fundamental role in peptide binding and affect T‐cell recognition (Rossman et al., 2003).

Argininemia (OMIM #207800) is caused by the deficiency of the enzyme arginase (encoded by the gene *ARG1*, OMIM *608313) and is recessively inherited. The main symptom is the progressive accumulation of arginine and ammonia in the blood. The nervous system is particularly sensitive to the effects of excess ammonia. Patients with this syndrome might also present with anorexia and vomiting (Diez‐Fernandez, Rüfenacht, Gemperle, Fingerhut, & Häberle, 2018).

Infantile hypophosphatasia (OMIM #241500) is a recessively inherited disorder that affects the development of bones and teeth. It is caused by mutations in the *ALPL* (OMIM *171760), which encodes an alkaline phosphatase that plays a key role in skeletal mineralization by regulating levels of the diphosphate. The signs and symptoms of hypophosphatasia vary widely. Hypophosphatasia weakens and softens the bones, causing skeletal abnormalities including short limbs, abnormally shaped chest, and soft skull bones. Additional complications include anorexia and recurrent vomiting (Weiss et al., 1988).

Cyclic vomiting syndrome (OMIM #500007) is a disorder characterized by recurrent episodes of nausea, vomiting, and lethargy. In 2003, a study on an Italian family identified a mutation (in the mitochondrial gene *MTTL1*, OMIM *590050) co‐segregating with the phenotype in three subjects. This gene is transcribed in the leucine mitochondrial transfer RNA. The presence of recurrent vomiting and nausea can mimic the AN (Haan, Kors, & Ferrari, 2002).

Distal renal tubular acidosis with hemolytic anemia (OMIM: #611590) is a condition in which the kidneys are unable to remove enough acid from the body, making the blood too acidic. The inability to remove acids from the body results in delayed growth and softening of the bones; patients also show anorexia among their symptoms. This syndrome is caused by biallelic mutations in *SLC4A1* (OMIM +109270) that encodes a chloride/bicarbonate exchanger (Yenchitsomanus et al., 2003). This protein functions as a transporter that mediates electroneutral anion exchange across the cell membrane. It is expressed in the erythrocyte where it exchanges inorganic anions across the membrane, and in the kidney where it mediates the chloride‐bicarbonate exchange required for the normal acidification of the urine (Yenchitsomanus et al., 2003).

In 2015, a paper reported a 12‐year‐old female with severe anorexia and body weight loss, resembling the restricting AN. Consequently, she was first thought to have AN. However, a history of neonatal hepatitis led clinicians to suspect a citrin deficiency. Genetic analysis of *SLC25A13* (OMIM *603859) revealed two compound heterozygous pathogenic variants and the diagnosis of citrin deficiency was confirmed (Takeuchi et al., 2015).

### 3.4 Mouse models

Mouse models are particularly important for the study of the molecular basis of AN and several genes have been found to play a key role. Animal studies on rats have established that MC4R activation decreases food intake along with the activation of the hypothalamic‐pituitary adrenal (HPA) axis and, thus, increases motor activity. These studies strongly suggest that genetic defects leading to the prolonged activation of the melanocortinergic system can potentially lead to AN (Liu, Garza, Li, & Lu, 2013).

The *PMCH* (pro‐melanin‐concentrating hormone) (OMIM *176795) encodes a prohormone precursor of melanin‐concentrating hormone (MCH), which is a hypothalamic neuropeptide. The mouse KO of *PMCH* results in a decrease in food intake, body weight, and body adiposity (Georgescu et al., 2005; Shimada, Tritos, Lowell, Flier, & Maratos‐Flier, 1998). Consistently, transgenic mice with increased expression of PMCH have the opposite phenotype, with hyperphagia and mild obesity (Shimada et al., 1998).

The MCH1 receptor (MCH1R) is known to play a significant role in energy homeostasis by increasing locomotor activity, appetite, neuroendocrine function, and metabolic rate. MCH1R is a G protein‐coupled receptor that is responsible for MCH signal transduction. In rodents, the MCH receptor (MCH1R) is greatly expressed in the nucleus accumbens shell (AcSh), where it regulates appetite (Georgescu et al., 2005). Mch1r−/− mice have a body weight within the normal range, but are lean and have a reduced fat mass. Interestingly, Mch1r−/− mice are lean because of hyperactivity and altered metabolism (Marsh et al., 2002).

KO mice for the M3 muscarinic receptor gene *Chrm3* (Chrm3–/–) have decreased body weight and fat, as well as hypophagia. Even when they are injected with orexigenic agents like AGRP, they maintain a reduced feeding capacity (Yilmaz et al., 2015).

*Cnr1* null mice show a significant bodyweight reduction due to reduction in food intake, although they are fed with a standard chow diet and are resistant to high‐fat obesogenic diets (Yilmaz et al., 2015).

OPRD1 KO mice (Oprd1–/–) are resistant to weight gain induced by a high‐fat diet because of increased thermogenesis. Consistent with this, several studies that reported single nucleotide variants in *OPRD1* were associated with AN in humans (Czyzyk et al., 2012).

Inactivation of the *Htr4* in mice causes suppression of motor hyperactivity. Consistently, stimulation of *Htr4* triggers hyperactivity and anorexia. These mice have a high rewarding effect of hyperactivity and food restriction, so that they tend to eat less and ultimately develop AN (Jean et al., 2012).

The *CYFIP1* (cytoplasmic FMR1‐interacting protein 1) (OMIM *606322) is involved in neurodevelopmental disorders. It was reported that a microduplication of the locus 15q11.2 that encompasses the *CYFIP1* is significantly associated with AN (Chang et al., 2019). Not surprisingly, *CYFIP1* is one of the four paternally deleted genes in patients with type I Prader–Willi syndrome, a neurodevelopmental disorder. Type I Prader–Willi syndrome symptoms include hyperphagia, obesity, cognitive deficits, and obsessive–compulsive behaviors. Interestingly, *Cyfip1* haploinsufficiency in mice increased compulsive‐like behavior and induced genetic background‐dependent, sex‐dependent, and parent‐of‐origin‐dependent increases in palatable food intake (Babbs et al., 2019). See Table 2 for a summary of genes mutated in mice models for AN.

**Table 2:** Genes that when knocked‐out cause anorexia in mice. (www.informatics.jax.org/)

Human gene	Gene function	Knocked‐out mouse gene	Mouse features	Reference or MGI ID
*PMCH*	Melanin‐concentrating hormone neuropeptide	*Pmch*	Decrease of food intake, body weight, body adiposity	Shimada et al. (1998)
*CHRM3*	Regulator of glucose homeostasis by modulating insulin secretion	*Chrm3*	Decrease of body weight and fat mass, hypophagia	Yilmaz et al. (2015)
*CNR1*	Orexigenic, regulation of energy expenditure	*Cnr1*	Reduction of food intake and body weight	Yilmaz et al. (2015)
*OPRD1*	Receptor for endogenous opioids	*Oprd1*	Resistance to weight gain, increased thermogenesis, leanness	Czyzyk et al. (2012)
*NPY2R* *NPY4R*	Orexigenic	*Npy2r* *Npy4r*	Reduction of adipose mass, increase of lean mass	108418 105374
*HCRT*	Regulation of food intake and sleep‐wakefulness	*Hcrt*	Decreased food and water intake	1,202,306
*HTR4* *HTR1B* *HTR1A*	Serotonin receptors	*Htr4* *Htr1B*	Voluntary food restriction following stress; reduction of novelty‐induced exploratory activity	Jean et al. (2012)
*DRD1* *DRD2*	Activation of pathways mediated by G protein‐coupled receptors	*Drd1* *Drd2*	Hypophagia, locomotor deficiency	99578 94924
*DAGLA* *DAGLB*	Biosynthesis of 2‐arachidonoylglycerol	*Dagla* *Daglb*	Hypophagia, decreased body weight, leanness	2677061 2442032
*NAPEPLD*	Biosynthesis of anandamide	*Napepld*	Hypophagia, decreased body weight, leanness	2140885
*CCK*	Regulation of pancreatic digestive enzymes	*Cck*	Impaired fat digestion and absorption	88297
*GLP1R*	Stimulation of glucose‐induced insulin secretion	*Glp1r*	Reduction of feeding behavior	99571
*CRH*	Release of hormones involved in the stress response	*Crh*	Inhibition of food intake	88496
*CYFIP1*	FMR1‐mediated translation repression in the brain	*Cyfip1*	Disordered eating	Babbs et al. (2019)

## 4 MOLECULAR DIAGNOSIS

With this paper, we intend to highlight the necessity and the urgency to develop a comprehensive genetic test for the diagnosis of AN. This need has been made clear in an editorial of The British Journal of Psychiatry (Curtis, Adlington, & Bhui, 2019). The authors clearly state that there is an increasing need to understand the genetic basis of several psychiatric disorders, including AN, to establish the causes of that condition and to provide information on the recurrence risk in the relatives of AN patients. Obviously, it is still difficult to arrive at a clear answer using a genetic test in cases like AN (Curtis et al., 2019).

In particular, we propose a test in which we would analyze genes involved in AN, subdivided into four groups. The first group of gene would encompass genes that have been found to carry rare variants in patients with AN for whom segregation analysis has been performed. In a second group, we would include genes associated with syndromes presenting anorexia among their main features. In a third set, we would analyze genes that have been found to carry polymorphisms conferring a higher susceptibility to the onset of AN. And finally, in the fourth group, we would include candidate genes for which mice models of AN are available.

## 5 DISCUSSION

Genetic testing will give several possible benefits for the diagnosis of AN: the establishment of the recurrence risk, identification of the pathways involved in the onset of the disease, and, in the future, identification of possible treatments on the basis of the genetic background (Curtis et al., 2019).

With the help of a genetic diagnostic test, most genes and SNPs involved in the primary and syndromic forms of AN could be analyzed in a single run, increasing our knowledge of the genetic background on which the environment and lifestyle factors might influence the onset of AN (Curtis et al., 2019).

Presently, there are no approved drug treatments for AN. Most psychoactive drugs only give symptomatic relief to some extent, but do not offer a cure for this condition. Therefore, new and tailored treatment options are required after proper genetic diagnosis, and those options could be tested in both in vitro and in vivo models to establish their efficacy and safety profiles. Molecules that can target specific pathways might be useful for targeted treatment of AN.

For example, cannabinoid receptors are known to link hedonic response with food‐related reward processes. Interestingly, AN patients have high plasma endocannabinoid levels that suggest an impairment in the endocannabinoid system (Andries & Støving, 2011). However, only one controlled trial was performed using a cannabinoid receptor agonist (tetrahydrocannabinol) in the therapy in AN patients, but this trial did not give significant results (Andries & Støving, 2011). Subsequently, numerous studies were performed in patients who suffer from anorexia due to other disorders, such as cancer or AIDS, that apparently demonstrated an increase in body weight. However, the mechanisms of the onset of that kind of anorexia are quite different (Andries & Støving, 2011).

Palmitoylethanolamide is synthesized from fatty acids and has been shown to bind cannabinoid receptors (Costa, Comelli, Bettoni, Colleoni, & Giagnoni, 2008). Possible use of palmitoylethanolamide in the treatment of AN was recently hypothesized. In fact, while several studies suggest that palmitoylethanolamide may lead to weight loss, possibly thanks to its anti‐inflammatory effects, these anti‐inflammatory function could stabilize hypothalamic responsiveness, that, in turn, causes weight loss when the patient is obese, and weight gain when the patient is anorexic.

Another possible treatment for AN could be the use of antagonists of serotonin receptors. For instance, it is known that the stimulation of the 5‐HT4Rs activity in the brain nucleus accumbens reduces food intake and increases motor hyperactivity (Jean et al., 2012). This is consistent with the observation that anorexia and motor hyperactivity are both major features of AN, suggesting a possible involvement of 5‐HT4R dysfunction in the condition. Therefore, drugs that would inhibit the activity of 5‐HT4R would be potentially useful for the treatment of AN (Leibowitz, 1990).

1,25‐dihydroxyvitamin D3 is another compound that could be used for the treatment of AN. There are several studies that have found evidence for the role of 1,25‐dihydroxyvitamin D3 as a neuroprotective neurosteroid. 1,25‐dihydroxyvitamin D3 regulates calcium signaling in the brain by stimulating the expression of intracellular calcium‐binding proteins; it modulates the production of reactive oxygen species by increasing the concentrations of endogenous antioxidants in the brain and it stimulates the production of neurotrophic factors, such as the nerve growth factor, thus modulating neuronal survival and differentiation during development (Garcion et al., 1996; Ibi et al., 2001; Korsching, Auburger, Heumann, Scott, & Thoenen, 1985). 1,25‐dihydroxyvitamin D3 might also increase the production of glial cell‐derived neurotrophic factor, necessary for the development of the dopaminergic and noradrenergic systems (Oo & Burke, 1997; Quintero et al., 2004). Therefore, 1,25‐dihydroxyvitamin D3 can affect cellular development in brain regions whose abnormal function is believed to be central to various psychiatric conditions, such as AN.

Additionally, other studies evaluated the potential importance of amino acid supplementation in AN. Tryptophan supplementation in the treatment of AN patients was evaluated. Tryptophan is an essential amino acid and is the immediate precursor of 5‐HT (i.e., the neurotransmitter serotonin); it has been reported that food restriction and starvation decrease tryptophan as well as serotonin levels in the brain of AN patients. Studies have shown that tryptophan supplementation boosts serotonin neurotransmission, which leads to significant therapeutic effects in AN caused by serotonin deficiency (Haleem, 2017). Another study, performed in mice, showed that glutamine supplementation did not help to recover weight. However, it restores the intestinal barrier in the activity‐based anorexia mouse model (L’huillier et al., 2019).

Other compounds that could be tested in AN patients might be extracted from *Panax ginseng*. In fact, its extract, called ginsenoside, has positive effects on neurological disorders through its antioxidant and immunomodulatory properties. One study showed that, along with its antioxidant activity, ginsenoside also acts as a modulator of metabolism, intracellular signaling, mitochondrial function, and apoptosis genes (Kim et al., 2018). A research study has investigated the ginsenoside Rg1 effect on the ingestive behavior modulation and discovered that continuous administration of Rg1 diminished heat‐induced anorexia along with reduced ambulation and increased water intake during an environmental temperature increase. Similarly, constant infusion of Rg1, after surgically induced anorexia, prevented food intake suppression. This study showed that the rat model maintained its body weight, which indicates that constant Rg1 central administration relieves heat‐ and surgically induced anorexia (Fujimoto et al., 1989).

## 6 CONCLUSION

AN is a complex eating disorder in which various biological, genetic, and environmental factors contribute to the disease etiology. Hence, the relative risk for the disease susceptibility is higher in family members of AN patients than in the normal population. Candidate gene studies, as well as genome‐wide association studies, have established associations of many genetic variants with AN. Animal models are used to better understand the mechanism of onset of AN. Genome‐wide and candidate gene association studies, linkage analysis, and molecular diagnosis based on gene panels, whole exome, and genome sequencing will help to elucidate the complex genetic basis of AN and the many metabolic and molecular pathways altered in this condition, eventually providing more effective “tailored” therapeutic options for treating AN patients.

## CONFLICT OF INTEREST

The authors declare that there is no conflict of interest regarding the publication of this paper.

## AUTHORS CONTRIBUTION

SP contributed to the acquisition of literature data, critically revised the first draft, and wrote the final version of the manuscript; AKK contributed to the acquisition of literature data and wrote the first draft of the manuscript; EM, TB, MRC, LS, PC, and LDR contributed to acquisition of literature data and critically revised the manuscript; MB contributed to the conception of the work, acquisition of literature data, drafted, and critically revised the manuscript. All authors approved the final version to be published.

## References

[mgg31244-bib-0001] Abdalla, M. M. I. (2017). Central and peripheral control of food intake. Endocrine Regulations, 51(1), 52–70. 10.1515/enr-2017-0006 28222022

[mgg31244-bib-0002] Anand, B. , & Brobeck, J. (1951). Hypothalamic control of food intake in rats and cats. The Yale Journal of Biology and Medicine, 24(2), 123–140. 14901884 PMC2599116

[mgg31244-bib-0003] Andries, A. , & Støving, R. K. (2011). Cannabinoid‐1 receptor agonists: A therapeutic option in severe, chronic anorexia nervosa? Neuropsychiatry, 1(5), 467–476. 10.2217/npy.11.50

[mgg31244-bib-0004] Arcelus, J. , Mitchell, A. J. , Wales, J. , & Nielsen, S. (2011). Mortality rates in patients with anorexia nervosa and other eating disorders: A meta‐analysis of 36 studies. Archives of General Psychiatry, 68(7), 724–731. 10.1001/archgenpsychiatry.2011.74 21727255

[mgg31244-bib-0005] Babbs, R. K. , Beierle, J. A. , Ruan, Q. T. , Kelliher, J. C. , Chen, M. M. , Feng, A. X. , … Bryant, C. D. (2019). CyFIP1 haploinsufficiency increases compulsive‐like behavior and modulates palatable food intake in mice: Dependence on CYFIP2 genetic background, parent‐of origin, and sex. *G3: Genes, Genomes* . Genetics, 9(9), 3009–3022. 10.1534/g3.119.400470 PMC6723122 31324746

[mgg31244-bib-0006] Beck, B. (2006). Neuropeptide Y in normal eating and in genetic and dietary‐induced obesity. Philosophical Transactions of the Royal Society B: Biological Sciences, 361(1471), 1159–1185. 10.1098/rstb.2006.1855 PMC1642692 16874931

[mgg31244-bib-0007] Bello, V. , Moreau, N. , Sirour, C. , Hidalgo, M. , Buisson, N. , & Darribère, T. (2015). The dystroglycan: Nestled in an adhesome during embryonic development. Developmental Biology, 401(1), 132–142. 10.1016/j.ydbio.2014.07.006 25050932

[mgg31244-bib-0008] Bergen, A. W. , van den Bree, M. B. M. , Yeager, M. , Welch, R. , Ganjei, J. K. , Haque, K. , … Kaye, W. H. (2003). Candidate genes for anorexia nervosa in the 1p33‐36 linkage region: Serotonin 1D and delta opioid receptor loci exhibit significant association to anorexia nervosa. Molecular Psychiatry, 8(4), 397–406. 10.1038/sj.mp.4001318 12740597

[mgg31244-bib-0009] Bergen, A. W. , Yeager, M. , Welch, R. A. , Haque, K. , Ganjei, J. K. , van den Bree, M. B. M. , … Kaye, W. H. (2005). Association of multiple DRD2 polymorphisms with anorexia nervosa. Neuropsychopharmacology, 30(9), 1703–1710. 10.1038/sj.npp.1300719 15920508

[mgg31244-bib-0010] Berry, E. M. , & Mechoulam, R. (2002). Tetrahydrocannabinol and endocannabinoids in feeding and appetite. Pharmacology and Therapeutics, 95(2), 185–190. 10.1016/S0163-7258(02)00257-7 12182965

[mgg31244-bib-0011] Bienvenu, T. , Lebrun, N. , Clarke, J. , Duriez, P. , Gorwood, P. , & Ramoz, N. (2019a). De novo deleterious variants that may alter the dopaminergic reward pathway are associated with anorexia nervosa. Eating and Weight Disorders, 1–8, 10.1007/s40519-019-00802-9 31664672

[mgg31244-bib-0012] Bienvenu, T. , Lebrun, N. , Clarke, J. , Duriez, P. , Gorwood, P. , & Ramoz, N. (2019b). Exome sequencing in a familial form of anorexia nervosa supports multigenic etiology. Journal of Neural Transmission, 126(11), 1505–1511. 10.1007/s00702-019-02056-2 31388831

[mgg31244-bib-0013] Brown, K. M. O. , Bujac, S. R. , Mann, E. T. , Campbell, D. A. , Stubbins, M. J. , & Blundell, J. E. (2007). Further evidence of association of OPRD1 & HTR1D polymorphisms with susceptibility to anorexia nervosa. Biological Psychiatry, 61(3), 367–373. 10.1016/j.biopsych.2006.04.007 16806108

[mgg31244-bib-0014] Ceccarini, M. , Tasegian, A. , Franzago, M. , Patria, F. , Albi, E. , Codini, M. , … Beccari, T. (2019). 5‐HT2AR and BDNF gene variants in eating disorders susceptibility. American Journal of Medical Genetics. Part B, Neuropsychiatric Genetics. 10.1002/ajmg.b.32771 31746551

[mgg31244-bib-0015] Chang, X. , Qu, H. , Liu, Y. , Glessner, J. , Hou, C. , Wang, F. , … Hakonarson, H. (2019). Microduplications at the 15q11.2 BP1–BP2 locus are enriched in patients with anorexia nervosa. Journal of Psychiatric Research, 113, 34–38. 10.1016/j.jpsychires.2019.01.021 30878790 PMC6486445

[mgg31244-bib-0016] Chao, J. , & Nestler, E. J. (2004). Molecular neurobiology of drug addiction. Annual Review of Medicine, 55, 113–132. 10.1146/annurev.med.55.091902.103730 14746512

[mgg31244-bib-0017] Chen, J. , Kang, Q. , Jiang, W. , Fan, J. , Zhang, M. , Yu, S. , & Zhang, C. (2015). The 5‐HTTLPR confers susceptibility to anorexia nervosa in Han Chinese: Evidence from a case‐control and family‐based study. PLoS ONE, 10(3), e0119378 10.1371/journal.pone.0119378 25785698 PMC4364880

[mgg31244-bib-0018] Clarke, T. K. , Weiss, A. R. D. , & Berrettini, W. H. (2012). The genetics of anorexia nervosa. Clinical Pharmacology and Therapeutics, 91(2), 181–188. 10.1038/clpt.2011.253 22190067

[mgg31244-bib-0019] Compan, V. (2017). Serotonin 4 receptors: A cornerstone in anorexia nervosa? Autism‐Open Access, 17(2), 1–6. 10.4172/2165-7890.1000207

[mgg31244-bib-0020] Costa, B. , Comelli, F. , Bettoni, I. , Colleoni, M. , & Giagnoni, G. (2008). The endogenous fatty acid amide, palmitoylethanolamide, has anti‐allodynic and anti‐hyperalgesic effects in a murine model of neuropathic pain: Involvement of CB1, TRPV1 and PPARγ receptors and neurotrophic factors. Pain, 139(3), 541–550. 10.1016/j.pain.2008.06.003 18602217

[mgg31244-bib-0021] Cui, H. , Moore, J. , Ashimi, S. S. , Mason, B. L. , Drawbridge, J. N. , Han, S. , … Lutter, M. (2013). Eating disorder predisposition is associated with ESRRA and HDAC4 mutations. Journal of Clinical Investigation, 123(11), 4706–4713. 10.1172/JCI71400 24216484 PMC3809805

[mgg31244-bib-0022] Cummings, D. E. , & Overduin, J. (2007). Gastrointestinal regulation of food intake. Journal of Clinical Investigation, 117(1), 13–23. 10.1172/JCI30227 17200702 PMC1716217

[mgg31244-bib-0023] Curtis, D. , Adlington, K. , & Bhui, K. S. (2019). Pursuing parity: Genetic tests for psychiatric conditions in the UK National Health Service. British Journal of Psychiatry, 214(5), 248–250. 10.1192/bjp.2019.48 30900968

[mgg31244-bib-0024] Czyzyk, T. A. , Romero‐Picó, A. , Pintar, J. , McKinzie, J. H. , Tschöp, M. H. , Statnick, M. A. , & Nogueiras, R. (2012). Mice lacking δ‐opioid receptors resist the development of diet‐induced obesity. FASEB Journal, 26(8), 3483–3492. 10.1096/fj.12-208041 22593549 PMC3405260

[mgg31244-bib-0025] Dardennes, R. M. , Zizzari, P. , Tolle, V. , Foulon, C. , Kipman, A. , Romo, L. , … Epelbaum, J. (2007). Family trios analysis of common polymorphisms in the obestatin/ghrelin, BDNF and AGRP genes in patients with anorexia nervosa: Association with subtype, body‐mass index, severity and age of onset. Psychoneuroendocrinology, 32(2), 106–113. 10.1016/j.psyneuen.2006.11.003 17197106

[mgg31244-bib-0026] De Silva, A. , & Bloom, S. R. (2012). Gut hormones and appetite control: A focus on PYY and GLP‐1 as therapeutic targets in obesity. Gut and Liver, 6(1), 10–20. 10.5009/gnl.2012.6.1.10 22375166 PMC3286726

[mgg31244-bib-0027] Devlin, B. , Jones, B. L. , Bacanu, S. A. , & Roeder, K. (2002). Mixture models for linkage analysis of affected sibling pairs and covariates. Genetic Epidemiology, 22(1), 52–65. 10.1002/gepi.1043 11754473

[mgg31244-bib-0028] Diez‐Fernandez, C. , Rüfenacht, V. , Gemperle, C. , Fingerhut, R. , & Häberle, J. (2018). Mutations and common variants in the human arginase 1 (ARG1) gene: Impact on patients, diagnostics, and protein structure considerations. Human Mutation, 39(8), 1029–1050. 10.1002/humu.23545 29726057

[mgg31244-bib-0029] Di Guida, F. (2017). Pharmacological effects of palmitoylethanolamide on hypertension, insulin‐resistance and obesity in murine models. 10.6093/UNINA/FEDOA/11611.

[mgg31244-bib-0030] Dincheva, I. , Drysdale, A. T. , Hartley, C. A. , Johnson, D. C. , Jing, D. , King, E. C. , … Lee, F. S. (2015). FAAH genetic variation enhances fronto‐amygdala function in mouse and human. Nature Communications, 6, 6395 10.1038/ncomms7395 PMC4351757 25731744

[mgg31244-bib-0031] Doris, J. M. , Millar, S. A. , Idris, I. , & O’Sullivan, S. E. (2019). Genetic polymorphisms of the endocannabinoid system in obesity and diabetes. Diabetes, Obesity and Metabolism, 21(2), 382–387. 10.1111/dom.13504 30129173

[mgg31244-bib-0032] Fujimoto, K. , Sakata, T. , Ishimaru, T. , Etou, H. , Ookuma, K. , Kurokawa, M. , & Machidori, H. (1989). Attenuation of anorexia induced by heat or surgery during sustained administration of ginsenoside Rg1 into rat third ventricle. Psychopharmacology (Berl), 99(2), 257–260. 10.1007/BF00442819 2508164

[mgg31244-bib-0033] Garcion, E. , Do Thanh, X. , Bled, F. , Teissier, E. , Dehouck, M. P. , Rigault, F. , … Darcy, F. (1996). 1,25‐dihydroxyvitamin D3 regulates γ‐glutamyl transpeptidase activity in rat brain. Neuroscience Letters, 216(3), 183–186. 10.1016/0304-3940(96)87802-5 8897488

[mgg31244-bib-0034] Genis‐Mendoza, A. D. , Ruiz‐Ramos, D. , López‐Narvaez, M. L. , Tovilla‐Zárate, C. A. , Rosa García, A. , Cortes Meda, G. , … Nicolini, H. (2019). Genetic association analysis of 5‐HTR2A gene variants in eating disorders in a Mexican population. Brain and Behavior, 9(7), e01286 10.1002/brb3.1286 31199591 PMC6625474

[mgg31244-bib-0035] Georgescu, D. , Sears, R. M. , Hommel, J. D. , Barrot, M. , Bolaños, C. A. , Marsh, D. J. , … DiLeone, R. J. (2005). The hypothalamic neuropeptide melanin‐concentrating hormone acts in the nucleus accumbens to modulate feeding behavior and forced‐swim performance. Journal of Neuroscience, 25(11), 2933–2940. 10.1523/JNEUROSCI.1714-04.2005 15772353 PMC6725126

[mgg31244-bib-0036] Giollo, A. , Idolazzi, L. , Caimmi, C. , Fassio, A. , Bertoldo, F. , Dalle Grave, R. , … Gatti, D. (2017). Vitamin D levels strongly influence bone mineral density and bone turnover markers during weight gain in female patients with anorexia nervosa. International Journal of Eating Disorders, 50(9), 1041–1049. 10.1002/eat.22731 28593655

[mgg31244-bib-0037] Goodfellow, C. E. , & Glass, M. (2009). Anandamide receptor signal transduction. Vitamins and Hormones, 81, 79–110. 10.1016/S0083-6729(09)81004-2 19647109

[mgg31244-bib-0038] Gratacòs, M. , González, J. R. , Mercader, J. M. , de Cid, R. , Urretavizcaya, M. , & Estivill, X. (2007). Brain‐derived neurotrophic factor Val66Met and psychiatric disorders: Meta‐analysis of case‐control studies confirm association to substance‐related disorders, eating disorders, and schizophrenia. Biological Psychiatry, 61(7), 911–922. 10.1016/j.biopsych.2006.08.025 17217930

[mgg31244-bib-0039] Grice, D. E. , Halmi, K. A. , Fichter, M. M. , Strober, M. , Woodside, D. B. , Treasure, J. T. , … Berrettini, W. H. (2002). Evidence for a susceptibility gene for anorexia nervosa on chromosome 1. American Journal of Human Genetics, 70(3), 787–792. 10.1086/339250 11799475 PMC384957

[mgg31244-bib-0040] Groves, N. J. , McGrath, J. J. , & Burne, T. H. J. (2014). Vitamin D as a neurosteroid affecting the developing and adult brain. Annual Review of Nutrition, 34, 117–141. 10.1146/annurev-nutr-071813-105557 25033060

[mgg31244-bib-0041] Haan, J. , Kors, E. E. , & Ferrari, M. D. (2002). Familial cyclic vomiting syndrome. Cephalalgia, 22(7), 552–554. 10.1046/j.1468-2982.2002.00420.x 12230597

[mgg31244-bib-0042] Haleem, D. J. (2017). Improving therapeutics in anorexia nervosa with tryptophan. Life Sciences, 178, 87–93. 10.1016/j.lfs.2017.04.015 28438641

[mgg31244-bib-0043] Hansen, H. S. , Kleberg, K. , & Hassing, H. A. (2015). Non‐endocannabinoid N‐acylethanolamines and monoacylglycerols: old molecules new targets. In The Endocannabinoidome: The World of Endocannabinoids and Related Mediators (pp. 1–13). 10.1016/B978-0-12-420126-2.00001-8.

[mgg31244-bib-0044] Hao, S. , Avraham, Y. , Mechoulam, R. , & Berry, E. M. (2000). Low dose anandamide affects food intake, cognitive function, neurotransmitter and corticosterone levels in diet‐restricted mice. European Journal of Pharmacology, 392(3), 147–156. 10.1016/S0014-2999(00)00059-5 10762668

[mgg31244-bib-0045] Hasan, T. F. , & Hunaid, H. (2011). Anorexia nervosa: A unified neurological perspective. International Journal of Medical Sciences, 8(8), 679–703. 10.7150/ijms.8.679 22135615 PMC3204438

[mgg31244-bib-0046] Hong, S.‐H. , Lee, J.‐E. , An, S.‐M. , Shin, Y. Y. , Hwang, D. Y. , Yang, S. Y. , … An, B.‐S. (2017). Effect of vitamin D3 on biosynthesis of estrogen in porcine granulosa cells via modulation of steroidogenic enzymes. Toxicological Research, 33(1), 49–54. 10.5487/TR.2017.33.1.049 28133513 PMC5266376

[mgg31244-bib-0047] Ibi, M. , Sawada, H. , Nakanishi, M. , Kume, T. , Katsuki, H. , Kaneko, S. , … Akaike, A. (2001). Protective effects of 1α,25‐(OH)2D3 against the neurotoxicity of glutamate and reactive oxygen species in mesencephalic culture. Neuropharmacology, 40(6), 761–771. 10.1016/S0028-3908(01)00009-0 11369030

[mgg31244-bib-0048] Ishiguro, H. , Onaivi, E. S. , Horiuchi, Y. , Imai, K. , Komaki, G. , Ishikawa, T. , … Arinami, T. (2011). Functional polymorphism in the GPR55 gene is associated with anorexia nervosa. Synapse (New York, N. Y.), 65(2), 103–108. 10.1002/syn.20821 20506567

[mgg31244-bib-0049] Jean, A. , Laurent, L. , Bockaert, J. , Charnay, Y. , Dusticier, N. , Nieoullon, A. , … Compan, V. (2012). The nucleus accumbens 5‐HTR4‐CART pathway ties anorexia to hyperactivity. Translational Psychiatry, 2, e203 10.1038/tp.2012.131 23233022 PMC3565192

[mgg31244-bib-0050] Kalueff, A. V. , Lou, Y. R. , Laaksi, I. , & Tuohimaa, P. (2004). Increased anxiety in mice lacking vitamin D receptor gene. NeuroReport, 15(8), 1271–1274. 10.1097/01.wnr.0000129370.04248.92 15167547

[mgg31244-bib-0051] Kaye, W. H. , Ebert, M. H. , Gwirtsman, H. E. , & Weiss, S. R. (1984). Differences in brain serotonergic metabolism between nonbulimic and bulimic patients with anorexia nervosa. American Journal of Psychiatry, 141(12), 1598–1601. 10.1176/ajp.141.12.1598 6209991

[mgg31244-bib-0052] Kim, K. H. , Lee, D. , Lee, H. L. , Kim, C. E. , Jung, K. , & Kang, K. S. (2018). Beneficial effects of Panax ginseng for the treatment and prevention of neurodegenerative diseases: Past findings and future directions. Journal of Ginseng Research, 42(3), 239–247. 10.1016/j.jgr.2017.03.011 29989012 PMC6035378

[mgg31244-bib-0053] Klenotich, S. J. , Ho, E. V. , McMurray, M. S. , Server, C. H. , & Dulawa, S. C. (2015). Dopamine D 2/3 receptor antagonism reduces activity‐based anorexia. Translational Psychiatry, 5, e613 10.1038/tp.2015.109 26241351 PMC4564564

[mgg31244-bib-0054] Klump, K. L. , Miller, K. B. , Keel, P. K. , McGue, M. , & Iacono, W. G. (2001). Genetic and environmental influences on anorexia nervosa syndromes in a population‐based twin sample. Psychological Medicine, 31(4), 737–740. 10.1017/S0033291701003725 11352375

[mgg31244-bib-0055] Korsching, S. , Auburger, G. , Heumann, R. , Scott, J. , & Thoenen, H. (1985). Levels of nerve growth factor and its mRNA in the central nervous system of the rat correlate with cholinergic innervation. The EMBO Journal, 4(6), 1389–1393. 10.1002/j.1460-2075.1985.tb03791.x 2411537 PMC554356

[mgg31244-bib-0056] Kortegaard, L. S. , Hoerder, K. , Joergensen, J. , Gillberg, C. , & Kyvik, K. O. (2001). A preliminary population‐based twin study of self‐reported eating disorder. Psychological Medicine, 31(2), 361–365. 10.1017/S0033291701003087 11232922

[mgg31244-bib-0057] L’Huillier, C. , Jarbeau, M. , Achamrah, N. , Belmonte, L. , Amamou, A. , Nobis, S. , … Coëffier, M. (2019). Glutamine, but not branched‐chain amino acids, restores intestinal barrier function during activity‐based anorexia. Nutrients, 11(6), 1348 10.3390/nu11061348 PMC6628073 31208031

[mgg31244-bib-0058] Leibowitz, S. F. (1990). The role of serotonin in eating disorders. Drugs, 39(Suppl 3), 33–48. 10.2165/00003495-199000393-00005 2197074

[mgg31244-bib-0059] Li, D. , Chang, X. , Connolly, J. J. , Tian, L. , Liu, Y. , Bhoj, E. J. , … Hakonarson, H. (2017). A genome‐wide association study of anorexia nervosa suggests a risk locus implicated in dysregulated leptin signaling. Scientific Reports, 7(1), 3847 10.1038/s41598-017-01674-8 28630421 PMC5476671

[mgg31244-bib-0060] Liu, J. , Garza, J. C. , Li, W. , & Lu, X. Y. (2013). Melanocortin‐4 receptor in the medial amygdala regulates emotional stress‐induced anxiety‐like behaviour, anorexia and corticosterone secretion. International Journal of Neuropsychopharmacology, 16(1), 105–120. 10.1017/S146114571100174X 22176700 PMC3708461

[mgg31244-bib-0061] Lokuge, S. , Frey, B. N. , Foster, J. A. , Soares, C. N. , & Steiner, M. (2011). Depression in women: Windows of vulnerability and new insights into the link between estrogen and serotonin. Journal of Clinical Psychiatry, 72(11), e1563–e1569. 10.4088/JCP.11com07089 22127200

[mgg31244-bib-0062] Lombardi, L. , Blanchet, C. , Poirier, K. , Lebrun, N. , Ramoz, N. , Rose Moro, M. , … Bienvenu, T. (2019). Anorexia nervosa is associated with Neuronatin variants. Psychiatric Genetics, 29(4), 103–110. 10.1097/YPG.0000000000000224 30933048

[mgg31244-bib-0063] Lutter, M. , Bahl, E. , Hannah, C. , Hofammann, D. , Acevedo, S. , Cui, H. , … Michaelson, J. J. (2017). Novel and ultra‐rare damaging variants in neuropeptide signaling are associated with disordered eating behaviors. PLoS ONE, 12(8), e0181556 10.1371/journal.pone.0181556 28846695 PMC5573281

[mgg31244-bib-0064] Marsh, D. J. , Weingarth, D. T. , Novi, D. E. , Chen, H. Y. , Trumbauer, M. E. , Chen, A. S. , … Qian, S. (2002). Melanin‐concentrating hormone 1 receptor‐deficient mice are lean, hyperactive, and hyperphagic and have altered metabolism. Proceedings of the National Academy of Sciences of the United States of America, 99(5), 3240–3245. 10.1073/pnas.052706899 11867747 PMC122503

[mgg31244-bib-0065] Di Marzo, V. , Goparaju, S. K. , Wang, L. , Liu, J. , Bátkai, S. , Járai, Z. , … Kunos, G. (2001). Leptin‐regulated endocannabinoids are involved in maintaining food intake. Nature, 410(6830), 822–825. 10.1038/35071088 11298451

[mgg31244-bib-0066] Mercader, J. M. , RibasÃ©s, M. , GratacÃ²s, M. , GonzÃ¡lez, J. R. , BayÃ©s, M. , de Cid, R. , … Estivill, X. (2007). Altered brain‐derived neurotrophic factor blood levels and gene variability are associated with anorexia and bulimia. Genes, Brain and Behavior, 6(8), 706–716. 10.1111/j.1601-183X.2007.00301.x 17376155

[mgg31244-bib-0067] Miller, G. (2017). Appetite regulation: Hormones, peptides, and neurotransmitters and their role in obesity. American Journal of Lifestyle Medicine, 13(6), 586–601. 10.1177/1559827617716376 31662725 PMC6796227

[mgg31244-bib-0068] Monteleone, A. M. , Di Marzo, V. , Aveta, T. , Piscitelli, F. , Dalle Grave, R. , Scognamiglio, P. , … Maj, M. (2015). Deranged endocannabinoid responses to hedonic eating in underweight and recently weight‐restored patients with anorexia nervosa. American Journal of Clinical Nutrition, 101(2), 262–269. 10.3945/ajcn.114.096164 25646322

[mgg31244-bib-0069] Monteleone, P. , Bifulco, M. , Di Filippo, C. , Gazzerro, P. , Canestrelli, B. , Monteleone, F. , … Maj, M. (2009). Association of CNR1 and FAAH endocannabinoid gene polymorphisms with anorexia nervosa and bulimia nervosa: Evidence for synergistic effects. Genes, Brain and Behavior, 8(7), 728–732. 10.1111/j.1601-183X.2009.00518.x 19659925

[mgg31244-bib-0070] Monteleone, P. , Matias, I. , Martiadis, V. , De Petrocellis, L. , Maj, M. , & Di Marzo, V. (2005). Blood levels of the endocannabinoid anandamide are increased in anorexia nervosa and in binge‐eating disorder, but not in bulimia nervosa. Neuropsychopharmacology, 30(6), 1216–1221. 10.1038/sj.npp.1300695 15841111

[mgg31244-bib-0071] Moskowitz, L. , & Weiselberg, E. (2017). Anorexia nervosa/atypical anorexia nervosa. Current Problems in Pediatric and Adolescent Health Care, 47(4), 70–84. 10.1016/j.cppeds.2017.02.003 28532965

[mgg31244-bib-0072] Nakabayashi, K. , Komaki, G. , Tajima, A. , Ando, T. , Ishikawa, M. , Nomoto, J. , … Shirasawa, S. (2009). Identification of novel candidate loci for anorexia nervosa at 1q41 and 11q22 in Japanese by a genome‐wide association analysis with microsatellite markers. Journal of Human Genetics, 54(9), 531–537. 10.1038/jhg.2009.74 19680270

[mgg31244-bib-0073] Oo, T. F. , & Burke, R. E. (1997). The time course of developmental cell death in phenotypically defined dopaminergic neurons of the substantia nigra. Developmental Brain Research, 98(2), 191–196. 10.1016/S0165-3806(96)00173-3 9051260

[mgg31244-bib-0074] Perello, M. , & Dickson, S. L. (2015). Ghrelin signalling on food reward: A salient link between the gut and the mesolimbic system. Journal of Neuroendocrinology, 27(6), 424–434. 10.1111/jne.12236 25377898 PMC5033008

[mgg31244-bib-0075] Perry, B. , & Wang, Y. (2012). Appetite regulation and weight control: The role of gut hormones. Nutrition and Diabetes, 2, e26 10.1038/nutd.2011.21 23154682 PMC3302146

[mgg31244-bib-0076] Quintero, E. M. , Willis, L. M. , Zaman, V. , Lee, J. , Boger, H. A. , Tomac, A. , … Granholm, A.‐C. (2004). Glial cell line‐derived neurotrophic factor is essential for neuronal survival in the locus coeruleus‐hippocampal noradrenergic pathway. Neuroscience, 124(1), 137–146. 10.1016/j.neuroscience.2003.11.001 14960346

[mgg31244-bib-0077] Ribasés, M. , Gratacòs, M. , Fernández‐Aranda, F. , Bellodi, L. , Boni, C. , Anderluh, M. , … Estivill, X. (2005). Association of BDNF with restricting anorexia nervosa and minimum body mass index: A family‐based association study of eight European populations. European Journal of Human Genetics, 13(4), 428–434. 10.1038/sj.ejhg.5201351 15657604

[mgg31244-bib-0078] Rossman, M. D. , Thompson, B. , Frederick, M. , Maliarik, M. , Iannuzzi, M. C. , Rybicki, B. A. , … Monos, D. (2003). HLA‐DRB1*1101: A significant risk factor for sarcoidosis in blacks and whites. American Journal of Human Genetics, 73, 720–735. 10.1086/378097 14508706 PMC1180597

[mgg31244-bib-0079] Rueda, D. , Navarro, B. , Martínez‐Serrano, A. , Guzmán, M. , & Galve‐Roperh, I. (2002). The endocannabinoid anandamide inhibits neuronal progenitor cell differentiation through attenuation of the Rap1/B. Journal of Biological Chemistry, 277(48), 46645–46650. 10.1074/jbc.M206590200 12237305

[mgg31244-bib-0080] Sabir, M. S. , Haussler, M. R. , Mallick, S. , Kaneko, I. , Lucas, D. A. , Haussler, C. A. , … Jurutka, P. W. (2018). Optimal vitamin D spurs serotonin: 1,25‐dihydroxyvitamin D represses serotonin reuptake transport (SERT) and degradation (MAO‐A) gene expression in cultured rat serotonergic neuronal cell lines. Genes and Nutrition, 13, 19 10.1186/s12263-018-0605-7 30008960 PMC6042449

[mgg31244-bib-0081] Scott‐Van Zeeland, A. A. , Bloss, C. S. , Tewhey, R. , Bansal, V. , Torkamani, A. , Libiger, O. , … Schork, N. J. (2014). Evidence for the role of EPHX2 gene variants in anorexia nervosa. Molecular Psychiatry, 19(6), 724–732. 10.1038/mp.2013.91 23999524 PMC3852189

[mgg31244-bib-0082] Shah, M. , & Vella, A. (2014). Effects of GLP‐1 on appetite and weight. Reviews in Endocrine and Metabolic Disorders, 15(3), 181–187. 10.1007/s11154-014-9289-5 24811133 PMC4119845

[mgg31244-bib-0083] Shimada, M. , Tritos, N. A. , Lowell, B. B. , Flier, J. S. , & Maratos‐Flier, E. (1998). Mice lacking melanin‐concentrating hormone are hypophagic and lean. Nature, 396(6712), 670–674. 10.1038/25341 9872314

[mgg31244-bib-0084] Siegfried, Z. , Kanyas, K. , Latzer, Y. , Karni, O. , Bloch, M. , Lerer, B. , & Berry, E. M. (2004). Association study of cannabinoid receptor gene (CNR1) alleles and anorexia nervosa: Differences between restricting and bingeing/purging subtypes. American Journal of Medical Genetics, 125B(1), 126–130. 10.1002/ajmg.b.20089 14755457

[mgg31244-bib-0085] Sild, M. , & Booij, L. (2019). Histone deacetylase 4 (HDAC4): A new player in anorexia nervosa? Molecular Psychiatry, 24(10), 1425–1434. 10.1038/s41380-019-0366-8 30742020

[mgg31244-bib-0086] Södersten, P. , Nergårdh, R. , Bergh, C. , Zandian, M. , & Scheurink, A. (2008). Behavioral neuroendocrinology and treatment of anorexia nervosa. Frontiers in Neuroendocrinology, 29(4), 445–462. 10.1016/j.yfrne.2008.06.001 18602416

[mgg31244-bib-0087] Steiner, A. A. , & Romanovsky, A. A. (2007). Leptin: At the crossroads of energy balance and systemic inflammation. Progress in Lipid Research, 46(2), 89–107. 10.1016/j.plipres.2006.11.001 17275915 PMC1976277

[mgg31244-bib-0088] Strober, M. , Freeman, R. , Lampert, C. , Diamond, J. , & Kaye, W. (2000). Controlled family study of anorexia nervosa and bulimia nervosa: Evidence of shared liability and transmission of partial syndromes. American Journal of Psychiatry, 157(3), 393–401. 10.1176/appi.ajp.157.3.393 10698815

[mgg31244-bib-0089] Takeuchi, S. , Yazaki, M. , Yamada, S. , Fukuyama, T. , Inui, A. , Iwasaki, Y. , & Ikeda, S. I. (2015). An adolescent case of citrin deficiency with severe anorexia mimicking anorexia nervosa. Pediatrics, 136(2), e530–e534. 10.1542/peds.2014-4172 26195537

[mgg31244-bib-0090] Tasegian, A. , Curcio, F. , Dalla Ragione, L. , Rossetti, F. , Cataldi, S. , Codini, M. , … Albi, E. (2016). Hypovitaminosis D3, leukopenia, and human serotonin transporter polymorphism in anorexia nervosa and bulimia nervosa. Mediators of Inflammation, 2016, 8046479 10.1155/2016/8046479 26903713 PMC4745338

[mgg31244-bib-0091] Veronese, N. , Solmi, M. , Rizza, W. , Manzato, E. , Sergi, G. , Santonastaso, P. , … Correll, C. U. (2015). Vitamin D status in anorexia nervosa: A meta‐analysis. International Journal of Eating Disorders, 48(7), 803–813. 10.1002/eat.22370 25445242

[mgg31244-bib-0092] Wang, K. , Zhang, H. , Bloss, C. S. , Duvvuri, V. , Kaye, W. , Schork, N. J. , … Hakonarson, H. (2011). A genome‐wide association study on common SNPs and rare CNVs in anorexia nervosa. Molecular Psychiatry, 16(9), 949–959. 10.1038/mp.2010.107 21079607

[mgg31244-bib-0093] Watson, H. J. , Yilmaz, Z. , Thornton, L. M. , Hübel, C. , Coleman, J. R. I. , Gaspar, H. A. , … Bulik, C. M. (2019). Genome‐wide association study identifies eight risk loci and implicates metabo‐psychiatric origins for anorexia nervosa. Nature Genetics, 51(8), 1207–1214. 10.1038/s41588-019-0439-2 31308545 PMC6779477

[mgg31244-bib-0094] Weiss, M. J. , Cole, D. E. C. , Ray, K. , Whyte, M. P. , Lafferty, M. A. , Mulivor, R. A. , & Harris, H. (1988). A missense mutation in the human liver/bone/kidney alkaline phosphatase gene causing a lethal form of hypophosphatasia. Proceedings of the National Academy of Sciences of the United States of America, 85(20), 7666–7669. 10.1073/pnas.85.20.7666 3174660 PMC282253

[mgg31244-bib-0095] Wirz, L. , Reuter, M. , Felten, A. , & Schwabe, L. (2018). An endocannabinoid receptor polymorphism modulates affective processing under stress. Social Cognitive and Affective Neuroscience, 13(11), 1177–1189. 10.1093/scan/nsy083 30239920 PMC6234318

[mgg31244-bib-0096] Yenchitsomanus, P. T. , Sawasdee, N. , Paemanee, A. , Keskanokwong, T. , Vasuvattakul, S. , Bejrachandra, S. , … Promwong, C. (2003). Anion exchanger 1 mutations associated with distal renal tubular acidosis in the Thai population. Journal of Human Genetics, 48(9), 451–456. 10.1007/s10038-003-0059-6 12938018

[mgg31244-bib-0097] Yilmaz, Z. , Hardaway, A. , & Bulik, C. (2015). Genetics and epigenetics of eating disorders. Advances in Genomics and Genetics, 5, 131–150. 10.2147/agg.s55776 27013903 PMC4803116

[mgg31244-bib-0098] Zipfel, S. , Giel, K. E. , Bulik, C. M. , Hay, P. , & Schmidt, U. (2015). Anorexia nervosa: Aetiology, assessment, and treatment. The Lancet Psychiatry, 2(12), 1099–1111. 10.1016/S2215-0366(15)00356-9 26514083

